# Efficacy and safety of high and low dose recombinant human erythropoietin on neurodevelopment of premature infants: A meta-analysis: Retraction

**DOI:** 10.1097/MD.0000000000042733

**Published:** 2025-06-06

The article, “Efficacy and safety of high and low dose recombinant human erythropoietin on neurodevelopment of premature infants: A meta-analysis”,^1^ which appears in Volume 100, Issue 18 of *Medicine*, is being retracted over concerns with the methodology. After publication, concerns were raised over references to the “Begger test,” which does not exist in the literature and is indicative of possible fraud. Upon further investigation, authorship irregularities were also discovered in our submission system. As a result, the Publisher no longer has confidence in the integrity of the content or the authorship.
